# Cellular Senescence in Keloid Pathology: Mechanisms, Biomarkers, and Potential Therapeutic Targets

**DOI:** 10.3390/biomedicines14040912

**Published:** 2026-04-16

**Authors:** Yujiang Luo, Yaxiong Deng, Li Yuan, Siqi Fu

**Affiliations:** 1 Department of Dermatology, The Second Xiangya Hospital of Central South University, Hunan Key Laboratory of Medical Epigenomics, 139 Middle Renmin Road, Changsha 410011, China; luoyujiang0609@163.com (Y.L.);; 2 Department of Nuclear Medicine, The Third Xiangya Hospital of Central South University, Changsha 410013, China; 3 Key Laboratory of Dermatology, Anhui Medical University, Ministry of Education, Hefei 230032, China

**Keywords:** keloid, cellular senescence, senescence-associated secretory phenotype, senescence-resumed proliferation, therapeutic target

## Abstract

A keloid is a benign fibroproliferative cutaneous disorder characterized by excessive extracellular matrix deposition, which is driven by persistent fibroblast proliferation and aberrant wound healing. Its complex pathogenesis involves genetic susceptibility, chronic inflammation, mechanical tension and dysregulated cellular signaling, resulting in poor clinical efficacy and high recurrence rates. Cellular senescence has recently become a central focus in exploring keloid pathophysiology, offering a novel perspective for elucidating its initiation, progression and recurrence. This review systematically summarizes the biological roles of cellular senescence in keloid pathology: it elaborates on the basic concepts and core molecular features of cellular senescence, details the spatial heterogeneity of senescent cell accumulation, the activation and pathological effects of senescence-associated secretory phenotype (SASP), and clarifies the molecular link between senescence-resumed proliferation (SRP) and keloid recurrence and treatment resistance. It also summarizes advances in senescence-related markers, the regulatory roles of the p53/p21 and Wnt/β-catenin pathways, and potential senescence-targeted therapies (senolytic, senomorphic, signaling intervention, cell reprogramming). Finally, we discuss the challenges and future perspectives for translating senescence research into clinical keloid treatments, aiming to provide a novel theoretical framework and therapeutic targets for keloid management.

## 1. Introduction

Keloids are benign fibroproliferative skin lesions caused by dysregulated wound healing, characterized by persistent fibroblast activation and ECM accumulation [1,2,3]. A hallmark feature is their excessive proliferation and extension beyond the margins of the original wound [4]. Histologically, keloids exhibit abundant deposition of thick eosinophilic collagen bundles [5,6,7]. Persistent inflammatory signaling, particularly through the TGF-β/Smad and IL-6 pathways, sustains fibroblast activation and promotes their transdifferentiation into myofibroblasts, resulting in excessive collagen deposition and progressive fibrosis in keloids [8]. Although keloids are not life-threatening, their poor treatment response and high recurrence rates often cause cosmetic disfigurement and functional impairment. This imposes a heavy psychological burden and markedly reduces patients’ quality of life (QoL) [4]. In genetically susceptible individuals, keloids develop from dysregulated wound healing, which is characterized by persistent inflammation, aberrant fibroblast activation, and excessive ECM deposition [8,9,10]. Keloids are now recognized as a chronic inflammatory fibrotic disease [11].

Recent reviews have summarized that keloids develop from a complex interplay of multiple factors. Their high recurrence rate, chronic inflammation, and significant clinical burden support the reclassification of keloids as a distinct disease [4,7,8,11,12,13]. Accordingly, increasing attention has been directed toward the histopathological and immunological characteristics of keloids [14]. In recent years, the potential involvement of cellular senescence in keloid pathogenesis has attracted increasing interest. This review aims to explore the roles of cellular senescence in keloid initiation, progression, and recurrence, as well as its potential as a therapeutic target. Although cellular senescence limits fibroblast proliferation through growth arrest, SASP drives chronic inflammation and microenvironmental remodeling, thus contributing to pathological fibrosis [15,16,17]. Notably, SRP has been recently proposed as a novel mechanism in keloids, whereby senescent cells escape p21-mediated growth arrest and regain proliferative capacity [18]. To our knowledge, few integrative reviews have focused on cellular senescence in keloids. Elucidating the molecular mechanisms of keloid formation, particularly cellular senescence–associated pathways, may reveal novel therapeutic targets and improve clinical outcomes [19,20]. Therefore, this review focuses on summarizing recent advances in the understanding of cellular senescence in keloids, with the aim of providing new insights for clinicians and researchers.

## 2. Basic Concepts of Cellular Senescence

### 2.1. Cellular Senescence

Cellular senescence was first identified in cultured primary human fibroblasts by Hayflick and Moorhead in 1961. Cellular senescence is conventionally defined as a stable, mostly irreversible state of cell cycle arrest induced by various stressors, including telomere attrition, DNA damage, and oncogenic activation [21]. Emerging evidence indicates that the senescence definition is continuously evolving [22,23], characterizing senescence as a dynamic, heterogeneous process rather than a static endpoint [24]. Under specific molecular contexts, particularly before the establishment of a robust p16^INK4A^-mediated barrier, cells may bypass this growth arrest through the inhibition of p53 or pRb signaling pathways [25,26]. In keloids, cellular senescence acts as a double-edged sword. It serves as a vital barrier against cancer development. However, it also functions as an engine for chronic fibrosis by releasing inflammatory factors [27]. Some cells undergo ‘senescence escape’, where they remodel their phenotype and gain stem cell-like traits [28]. This leads to SRP, a main driver of keloid growth [28]. Furthermore, the morphological hallmarks of these states exhibit significant heterogeneity across different cell types, including fibroblasts and epithelial cells, reflecting the diverse cellular responses to pro-senescent stressors [29,30,31] (see Section 4 for a more detailed morphological hallmarks).

### 2.2. Cellular Senescence in Keloids

#### 2.2.1. Accumulation of Senescent Cells

**Figure 1:**
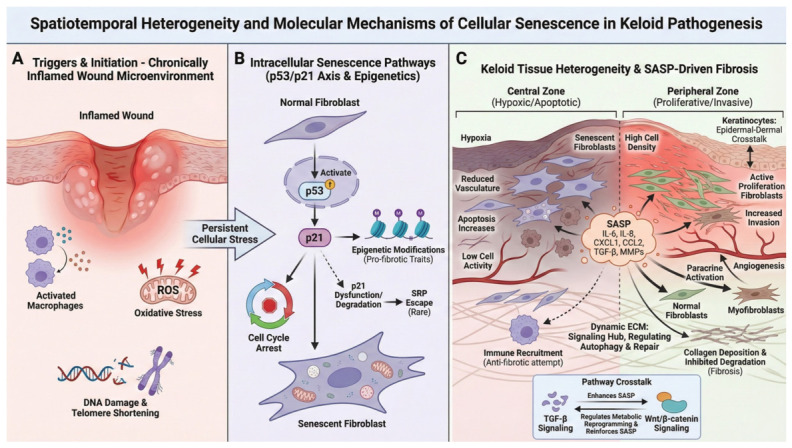
Cellular senescence drives spatiotemporal heterogeneity and fibrosis in keloids. (**A**) Chronic inflammatory wound stress (immune activation, ROS, DNA damage/telomere dysfunction) initiates senescence. The blue and orange dots represent pro-inflammatory cytokines secreted by activated macrophages. (**B**) Persistent stress engages intracellular senescence programs centered on the p53-p21 axis and epigenetic remodeling, enforcing stable fibroblast cell-cycle arrest. (**C**) Keloids display spatial heterogeneity, with senescent fibroblasts enriched in hypoxic central regions and proliferative/invasive fibroblasts in the periphery; senescent fibroblasts promote fibrosis via SASP-mediated paracrine signaling that enhances immune recruitment, fibroblast activation, angiogenesis, and extracellular matrix deposition, sustaining keloid progression. Created in BioRender. Luo, Y. (2026). BioRender.com/55otp0h.

Studies have identified distinct cellular senescence features between the central and peripheral regions of keloids [32]. The central region exhibits reduced cellular activity, poor vascularization, and increased apoptosis, whereas the peripheral region has high cell density, active proliferation, and enhanced invasiveness [33]. Importantly, senescent cell accumulation creates a local pro-senescent niche. This microenvironment drives aberrant fibroblast activation, thereby linking cellular senescence to keloid pathogenesis [33]. Chronic oxidative stress (excessive ROS generation), mechanical tension detected via mechanosensitive channels such as PIEZO2, and persistent TGF-β signaling may trigger senescence in keloid fibroblasts (Figure 1A; Section 3) [34]. Recent single-cell RNA sequencing (scRNA-seq) studies have identified at least four distinct subpopulations of keloid fibroblasts (KFs) [35,36]. These cells, characterized by aberrant dynamics and intricate crosstalk, synergistically drive keloid progression [37]. Single-cell transcriptomic and proteomic analyses have identified enriched senescence-associated marker-expressing fibroblasts in keloids [12,19]. These include upregulated p16^INK4a^, increased senescence-associated β-galactosidase (SA-β-Gal) positive cells, and a proinflammatory SASP profile (Figure 1B). The interplay between senescent cell accumulation and SASP secretion establishes a self-reinforcing ‘senescence-fibrosis’ loop, further exacerbating keloid formation (Figure 1C) [27,38].

Paralleling findings in pulmonary fibrosis highlight that senescent epithelial cells promote fibrosis via SASP-dependent activation of fibroblasts, which is tightly regulated by AKT/mTOR/NF-κB signaling [39]. Typically, senescence is initiated by DNA damage-induced DNA damage response (DDR) activation, which engages the p53/p21 and p16/Rb signaling pathways [40,41]. This persistent signaling converges on NF-κB and mTOR to facilitate SASP production [42]. Despite undergoing growth arrest, senescent cells may paradoxically promote keloid progression through sustained inflammatory signaling and microenvironmental remodeling [43,44]. By secreting profibrotic cytokines and growth factors, these cells alter the microenvironment and stimulate neighboring ‘non-senescent’ fibroblasts to hyper-proliferate and overproduce collagen, creating a self-perpetuating fibrotic loop [33]. Emerging evidence challenges the traditional view of senescence as a permanent endpoint. Specifically, it is hypothesized that cells with low p16^INK4a^ expression can bypass p21-mediated arrest to resume proliferation [25]. Upon DDR activation, p53 induces p21^CIP1^, which inhibits cyclin-dependent kinases (CDKs, e.g., CDK4/6, CDK2) and blocks the G1/S cell cycle transition. Similarly, p16^INK4a^ represses CDK4/6 to maintain inoblastoma protein (RB) in its active, hypophosphorylated state, thereby sustaining growth arrest. Senescent cells secrete the SASP, disrupting microenvironmental homeostasis (Figure 1B) [45]. Notably, the selective induction of apoptosis in KFs by the senolytic peptide FOXO4-DRI underscores the therapeutic potential of targeting senescence in keloids [19,20].

#### 2.2.2. SASP

It is well-established that the SASP, a hallmark of cellular senescence, comprises profibrotic and proinflammatory cytokines, chemokines, growth factors, and proteases that remodel the tissue microenvironment [30]. Chronic SASP signaling drives inflammation and fibrosis across diverse pathological settings [46,47].

In keloids, accumulating evidence suggests a senescence-associated inflammatory phenotype characterized by upregulated IL-6 and IL-8 expression (Figure 1A). This profile likely facilitates fibroblast activation and ECM remodeling [6,44]. These mediators crosstalk with core signaling pathways, such as TGF-β and Wnt/β-catenin, to perpetuate fibrotic progression [48].

At the molecular level, senescent cells exhibit activation of canonical markers, including p16 (CDKN2A), p21 (CDKN1A), and p53, which are induced downstream of persistent stress and DDR signaling. These cells acquire a hypersecretory phenotype (SASP), linking senescence to inflammation and tissue remodeling [46].

Mechanistically, SASP production is tightly regulated by signaling networks such as NF-κB and mTOR, which integrate stress signals and drive inflammatory gene expression [49,50]. These pathways amplify inflammatory signaling and contribute to a self-sustaining pro-fibrotic microenvironment.

#### 2.2.3. SRP

Emerging concepts define SRP as the phenomenon in which senescent cells regain their proliferative capacity [51]. Specifically, single-nucleotide variants (SNVs) in loci such as 1q41 and NEDD4 are associated with keloid susceptibility [52]. It is suggested that these variants predispose KFs to bypass p53-mediated growth checkpoints. In keloids, fibroblasts may escape senescence through epigenetic changes or mutations, resulting in abnormal proliferation of scar tissue [53,54]. This underscores the challenge in treating keloids, as SRP contributes to persistent growth and recurrence.

Furthermore, dynamic crosstalk between senescent cells and the keloid microenvironment acts as a critical driver of disease progression. Senescent fibroblasts alter the local environment through SASP, influencing immune cells, endothelial cells, and stem cell function, thus promoting chronic inflammation and sustaining fibrosis [19,55]. These interactions highlight the complexity of keloid pathology and suggest that therapies targeting SASP could modulate the local immune response and improve outcomes.

Understanding the role of cellular senescence in keloid formation not only sheds light on the underlying mechanisms but also provides a foundation for developing more effective therapeutic strategies [32]. Targeting SASP and addressing SRP may help prevent recurrence and improve treatment outcomes for keloid patients [55].

## 3. The Relationship Between Cellular Senescence and Keloids

**Figure 2:**
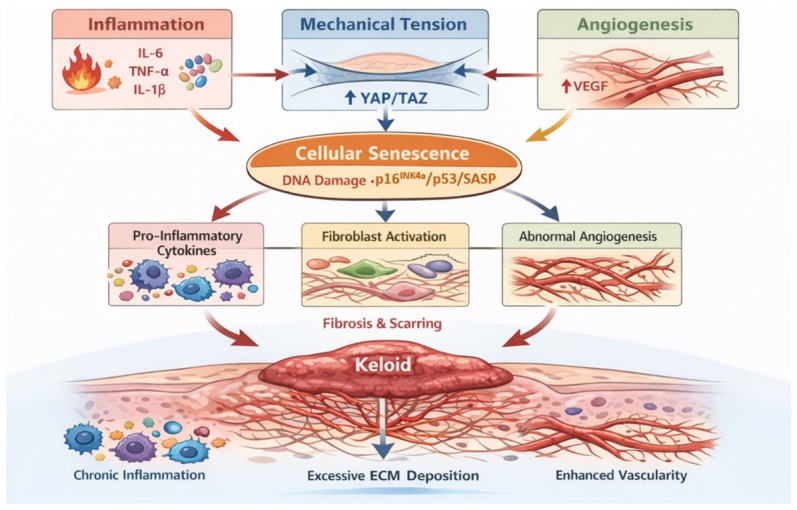
Mechanisms of Cellular Senescence in Keloid Formation: Inflammation, Mechanical Tension, and Angiogenesis. This infographic illustrates how the key factors of inflammation, mechanical tension, and angiogenesis induce cellular senescence (DNA damage, p16^INK4a^/p53/SASP) and contribute to keloid development. These factors promote fibroblast activation, abnormal angiogenesis, and excessive collagen deposition, leading to chronic inflammation and fibrosis. Molecules such as IL-6, tumor necrosis factor-alpha (TNF-α), vascular endothelial growth factor (VEGF), IL-1β, YAP/TAZ play pivotal roles in this process. Created in BioRender. Luo, Y. (2026) https://BioRender.com/3bue5i5/ (accessed on 20 February 2026).

While keloids are typically benign fibroproliferative lesions, chronic inflammation and recurrent ulceration create a highly stressful microenvironment that may occasionally induce malignant transformation [56,57,58,59]. Although emerging evidence indicates rare malignant transformation of keloid tissue [60], no definitive association between carcinoma and keloids has been established to date. Consequently, Liu et al. [61] proposed that dysregulated inflammation and proliferation in keloids create a ‘pro-tumor-like’ milieu. However, actual malignant transformation remains clinically rare [62]. Given the multifaceted regulatory network underlying keloid formation, Figure 2 illustrates how chronic microenvironmental stressors-namely prolonged inflammation, mechanical tension, and angiogenesis-trigger this senescent state [63]. These stressors may induce DNA damage and activate the *p*16^INK4a^ and p53 signaling pathways, leading to excessive SASP secretion [64]. Ultimately, this creates a vicious cycle that may continuously drives keloid progression (Figure 2).

### 3.1. Senescence-Associated Inflammation in Keloid Development

**Figure 3:**
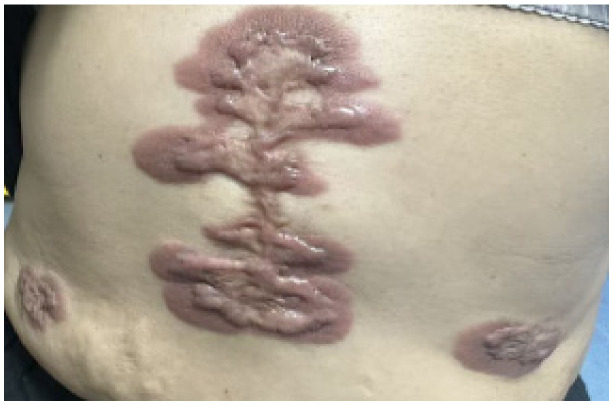
The hyperplastic halo surrounding the keloid protrusion tissue. Written informed consent for publication of this image was obtained from the patient.

Keloids may occasionally present with pruritus, pain, and a hyperplastic halo (Figure 3 for morphological features) [65,66]. Recurrent itch-pain cycles and persistent inflammatory mediator release indicate repeated stimulation, which drives the transition from transient to chronic inflammation [44]. There may be consistency between this observation and the concept that keloids are fibroproliferative disorders driven by persistent dermal inflammation. Notably, this persistent low-grade inflammation resembles ‘inflammaging’—a hallmark of aging defined by immune dysregulation and chronic cytokine production. This process is increasingly recognized as a contributor to multiple age-related pathologies [62,67,68,69]. In this context, cellular senescence and immune dysfunction contribute to a pro-inflammatory microenvironment through the secretion of SASP factors, including IL-6 and TNF-α, thereby reinforcing inflammation and tissue remodeling [70,71].

Mechanistically, the cyclic GMP-AMP synthase-stimulator of interferon genes (cGAS-STING) is an innate immune signaling pathway that senses cytosolic DNA [72]. The enzyme cGAS detects aberrant DNA in the cytoplasm and produces cyclic GMP–AMP (cGAMP), which activates STING, leading to the induction of type I interferons (type I IFN) and pro-inflammatory cytokines [73,74,75,76,77]. Similarly, cytoplasmic chromatin fragments generated during senescence engage the same cGAS–STING pathway, reinforcing sterile inflammatory signaling and SASP production [76,78]. In parallel, DDR signaling promotes inflammatory cytokine secretion, linking genomic instability to the non-cell-autonomous effects of senescence [79,80]. Consistent with this inflammatory dysregulation, soluble human leukocyte antigen E (sHLA-E) has been proposed as a potential biomarker for keloid risk and recurrence, while targeting the IL-13RA2/STAT6 axis may represent a therapeutic strategy [81,82,83,84]. Together, these processes may establish a self-reinforcing inflammation–senescence loop that sustains fibrosis and keloid progression (Figure 2) [70,71].

### 3.2. Mechanical Tension Modulates Cellular Senescence and Amplifies Fibrotic Responses

Keloids predominantly develop at high-tension anatomical sites [85]. This distribution underscores the hypothesis that persistent mechanical loading is not merely permissive but actively instructive in lesion evolution, utilizing mechanotransduction programs that drive profibrotic transcriptional outputs [86]. In KFs, the mechanosensitive Hippo pathway effectors YAP/TAZ are significantly upregulated [87]. Their inhibition attenuates KF proliferation, migration, and collagen production, identifying tension-responsive YAP/TAZ signaling as a critical driver of matrix accumulation in keloids [87]. In parallel, progressive matrix stiffening can itself promote senescence of activated mesenchymal cells, and senescent myofibroblasts can release paracrine signals that enhance fibroblast activation and collagen deposition, thereby converting a mechanical cue into a self-reinforcing profibrotic secretory niche [88]. Collectively, these data support a model in which mechanical tension and stiffness in keloid-prone skin potentiate YAP/TAZ-dependent fibroblast activation while also fostering (directly or indirectly) senescence-associated secretory programs that sustain fibroblast activation and extracellular matrix overproduction (Figure 2) [87]. A study identifies PIEZO2 as a critical pressure-sensing molecule in keloid pathology with a strong correlation between PIEZO2 and collagen production (COL1A2) [89].

### 3.3. Aberrant Angiogenesis and Cellular Senescence in Keloid Progression

Keloid lesions exhibit pronounced fibrovascular remodeling. Recent single-cell and spatial transcriptomic analyses have unraveled intimate spatial and signaling crosstalk between disease-associated fibroblasts and endothelial cells [38], suggesting the existence of an instructive fibrovascular niche rather than a secondary vascular response. Endothelial cells in keloids exhibit mesenchymal activation signatures with dysregulated TGF-β/Smad signaling, supporting endothelial phenotypic plasticity that may contribute to persistent matrix remodeling and maladaptive angiogenesis [38]. From a complementary perspective, cellular senescence provides a mechanistic framework for vascular dysfunction, as senescent endothelial cells can sustain a proinflammatory SASP capable of modulating VEGF-dependent angiogenic behavior and stromal activation [34]. Consistent with this notion, recent scRNA-seq studies have identified a mesenchymal-activated endothelial subset enriched in keloids with enhanced fibroblast–endothelial crosstalk, providing a potential cellular substrate through which senescence/SASP-associated inflammatory cues may couple to aberrant angiogenesis [90]. Collectively, these observations support a feed-forward model in which endothelial phenotypic dysregulation and fibroblast–endothelial interactions reinforce senescence-associated inflammatory signaling, thereby stabilizing a non-resolving, profibrotic microenvironment in keloids (Figure 2) [91].

## 4. Markers of Cellular Senescence

Mounting evidence indicates that keloid tissues harbor a significant accumulation of senescent fibroblasts [55,92,93]. This is evidenced by heightened SA-β-gal activity and the upregulation of canonical cell-cycle inhibitors, such as p16^INK4a^ and p21^CIP1^. Although senescent KFs undergo cell cycle arrest, they remain metabolically active and secrete a robust SASP (TGF-β, IL-6, and CXCL8) [64]. This SASP paracrinally induces hyperproliferation and ECM deposition in adjacent non-senescent KFs, thus driving keloid expansion. Senescent cells also develop a pro-inflammatory secretory phenotype (SASP), encompassing cytokines, chemokines, and proteases that modulate the tissue microenvironment [94]. Replicative senescence is driven by telomere shortening/ DDR, but also by oncogene activation and oxidative stress, and results in characteristic morphological and molecular changes, including enlarged flattened cell shape, chromatin reorganization, and increased activity of senescence-associated SA-β-Gal [95].

Notably, the SASP of senescent keloid fibroblasts contains high-mobility group box 1 (HMGB1) [48,96]. As a pro-inflammatory mediator, HMGB1 mediates paracrine senescence and immune cell recruitment, thereby amplifying the profibrotic microenvironment [48,96]. At the molecular level, cellular senescence in keloids is closely associated with mitochondrial dysfunction, oxidative stress and unresolved DDR [34,97]. Several cellular markers are involved in the senescence of keloid fibroblasts [96,98,99]. Direct evidence for aldo-keto reductase family 1 member C3 (AKR1C3) in keloids remains limited [100]. However, studies in other pathological contexts have confirmed that elevated AKR1C3 expression attenuates oxidative stress and enhances cell survival under stress conditions [101,102]. Collectively, these reciprocal microRNA expression patterns and cellular markers provide a molecular basis for identifying pathogenic fibroblast subpopulations and support the development of targeted, marker-based therapeutic strategies.

Investigating AKR1C3 expression in keloid tissues represents a critical unaddressed gap that could unveil novel diagnostic markers [100]. Together, these molecular signatures suggest that impaired apoptotic clearance of senescent fibroblasts contributes to the persistence of a proinflammatory, profibrotic microenvironment in keloids.

Together, these molecular signatures—including the upregulation of core senescence markers (p16^INK4a^, p21^CIP1^, SA-β-gal), SASP factors (TGF-β, IL-6, CXCL8, MMPs, HMGB1) and potential stress-resistant mediator AKR1C3—suggest that impaired apoptotic clearance of senescent fibroblasts, coupled with their active SASP secretion, sustains a pro-inflammatory and pro-fibrotic microenvironment (Table 1) [34,48].

## 5. Potential Therapeutic Targets

### 5.1. Targeting Senescent Cells and Senescence-Associated Plasticity

Keloid lesions exhibit markers of stable cell cycle arrest, including p16, p21, and SA-β-gal [6,104]. This accumulation has sparked interest in senolytic therapies designed to selectively induce apoptosis in senescent cells. Fundamentally, senolytic therapies exploit the unique survival dependencies of senescent cells to induce apoptosis, thereby eliminating persistent SASP production at its source. Furthermore, senolytic therapies such as dasatinib (D) and quercetin (Q) can effectively induce senescent cell death [55,105], but their efficacy is influenced by SASP composition and cellular context. The combination of D + Q has demonstrated the ability to reduce fibrotic burden in preclinical aging models, although its specific efficacy in keloids requires further validation [106,107,108,109,110,111]. However, strategies targeting the clearance of senescent cells have not yet been widely applied in keloid treatment and require further research to explore their potential therapeutic value [112,113]. On the other hand, avoiding the induction of senescence (rather than simply clearing senescent cells) might more effectively block the source of senescence-associated secretory responses, thereby restoring tissue homeostasis through comprehensive regulation of multiple signaling pathways like TP53, Wnt/β-catenin, and TGF-β. Conversely, some studies report that D + Q combination treatment accelerates aging in young female mice, yielding contradictory findings [114].

Currently, quercetin is already used as a health supplement and clinical skincare ingredient. In-depth research into its mechanism of action and potential targets in keloid treatment will provide an important scientific basis and clinical application prospects for developing new therapeutic strategies [107,115]. Furthermore, recent studies suggest that senescence may not be a terminal state; the concept of SRP has been proposed as a potential mechanism for keloid recurrence and expansion, though this remains an emerging area of research [18].

### 5.2. Targeting SASP and Its Regulatory Networks

The SASP is recognized as a potent driver of tissue remodeling and chronic inflammation [116]. In keloids, elevated SASP factors such as IL-6, IL-8, and TGF-β have been observed, which are proposed to maintain a pro-fibrotic microenvironment by activating neighboring fibroblasts [6,14]. Mechanistically, SASP is orchestrated by the NF-κB and mTOR signaling pathways [34]. While direct clinical evidence in keloids is emerging, pharmacological modulation using senomorphic agents (e.g., metformin, rapamycin) has shown promise in suppressing the secretome and attenuating fibrosis in various mesenchymal models [117].

### 5.3. Intervening in Senescence-Inducing Signals

#### 5.3.1. Wnt/β-Catenin Signaling Pathway

The Wnt/β-catenin signaling pathway is implicated in keloid formation (Table 2). This pathway is involved in keloid pathogenesis by regulating cell proliferation, differentiation, and extracellular matrix deposition. Research finds that activation of this pathway is associated with increased expression of downstream target genes such as *Cyclin D1* and *c-Myc*, which contribute to enhanced fibroblast proliferation. Furthermore, inhibiting the Wnt/β-catenin signaling pathway by targeting the Frizzled receptor with small interfering RNA (siRNA) has been shown to suppress the proliferation and migration of keloid fibroblasts. Therefore, the Wnt/β-catenin signaling pathway and its key regulators may represent potential therapeutic targets for keloid treatment [118,119].

#### 5.3.2. TGF-β Signaling Pathway

Keloid lesions exhibit elevated TGF-β1/2 and reduced TGF-β3 expression. TGF-β1 promotes fibroblast proliferation and excessive ECM deposition via Smad-dependent and -independent pathways, although its exact role in scarring remains debated [120]. Recently identified miR-3606-3p has been reported to be downregulated in fibrotic skin disorders and correlates with disease severity, with no clinical trials in keloid models to date. By directly targeting *GAB1*, *ITGAV*, and *TGFBR2*, this miRNA inhibits integrin/FAK, AKT/ERK, and TGF-β/Smad2/3 signaling to suppress fibroblast activation and fibrosis. In a humanized keloid model, miR-3606-3p reduces fibrosis, representing a promising therapeutic target [120]. TGF-β is a major factor regulating keloid formation and may induce cellular senescence by altering cell cycle-related gene expression. Notably, recent studies identify CSE-dependent Smad3 S-sulfhydration (Cys121) as a novel post-translational modification that negatively regulates Smad3’s pro-fibrotic function [121]. Validated in systemic sclerosis (SSc), this mechanism may also regulate abnormal keloid fibroblast activation, offering new targeted intervention insights.

#### 5.3.3. PI3K/AKT/mTOR Pathway

PI3K/AKT/mTOR hyperactivation in keloids is associated with enhanced fibroblast survival, invasion, and ECM production, making this axis an attractive disease-modifying therapeutic target rather than a purely downstream effector [122]. Accumulating evidence suggests that sunitinib effectively inhibits Akt/PI3K/mTOR signaling in keloid-derived fibroblasts, induces cell cycle arrest and apoptosis, reduces the expression of type I/III collagen, and promotes scar regression in a human keloid explant xenograft model [123]. This target-centric view also aligns with senomorphic concepts, because mTOR activity can promote SASP output, and mTOR inhibition (e.g., rapamycin) has been shown to restrain SASP programs in canonical senescence systems [50]. However, the long-term safety and target specificity of PI3K/AKT/mTOR pathway inhibition in keloid therapy remain to be elucidated [124]. Accordingly, PI3K/AKT/mTOR inhibition may complement senolytics by simultaneously dampening fibroblast pro-fibrotic programs and reducing SASP-like inflammatory reinforcement. Notably, by attenuating mTOR-linked stress signaling that can license SASP-like outputs, PI3K/AKT/mTOR inhibition may represent a senescence-informed adjunct and merits evaluation alongside senolytics in rational combination paradigms [125,126]. Furthermore, the high expression of circCOL5A1 is significantly correlated with excessive ECM deposition in keloid tissue [125,126]. These results suggest that the PI3K/Akt signaling pathway and its upstream regulators like circCOL5A1 may provide new directions for keloid treatment.

#### 5.3.4. p53/p21 Signaling Pathway

The p53/p21 signaling pathway is a critical regulator of the cell cycle and apoptosis. p53, as a tumor suppressor gene, can maintain genomic stability by regulating the cell cycle, inducing apoptosis, etc. In keloids, the expression of the p53 gene is significantly decreased, while the expression of its downstream WWP1 (an E3 ubiquitin ligase) increases. These changes are closely associated with keloid formation. WWP1 inhibits the transcriptional activity of nuclear p53 via ubiquitination modification, which is associated with decreased apoptosis and enhanced KF proliferation. Furthermore, p53 gene mutation or functional loss is considered one of the susceptibility factors for keloids [127], and its low expression in keloids may be associated with the high proliferative activity of KFs [128]. Studies demonstrated that the cell-cycle inhibitor p21 places cells under immune surveillance, acting as a biological timer for cell fate decisions [128,129,130]. These findings further implicate the p53/p21 pathway in keloid pathogenesis [128,129,130]. Upregulation of p53-pS15 and p16 sustains a senescent microenvironment in keloids [19], and FOXO4-DRI has shown potential to inhibit keloid aggressiveness and recurrence. This agent exhibits potent efficacy in preclinical models, while its clinical application awaits further trial investigation.

#### 5.3.5. Hedgehog-GLI1 Signaling Pathway

Additionally, the Hedgehog signaling pathway plays an important role in keloid pathogenesis [73]. The Hedgehog-GLI1 signaling pathway is critical for tissue development and repair, and its aberrant activation is associated with the development of various fibrotic diseases and tumors. It may also exacerbate the fibrotic process through interactions with other signaling pathways like TGF-β. Research on keloid patient-derived fibroblasts found that the Hedgehog pathway and its downstream transcription factor GLI1 are upregulated in these cells [131]. Using the Hedgehog pathway inhibitor vismodegib (a drug primarily used to treat basal cell carcinoma) has been shown to reduce the volume of keloid-like tissue, lower collagen deposition, and downregulate the expression of related fibrotic genes (such as COL1A1, α-SMA) in both in vitro and animal models [132]. Collectively, these signaling pathways interact to regulate KF proliferation, survival, and ECM deposition, ultimately driving keloid formation (Table 2).

**Table 2:** Mechanisms of Action and Intervention Potential of Key Signaling Pathways in Keloids.

Signaling Pathway	Core Mechanism of Action	Function in Keloids	Key Intervention Targets/Strategies	Literature Support
Wnt/β-catenin	Regulates cell proliferation, differentiation, and extracellular matrix (ECM) deposition. Upregulates target genes like Cyclin D1, c-Myc upon activation.	Promotes abnormal fibroblast proliferation and migration, a core pathway in keloid formation.	Target Frizzled receptor (e.g., using siRNA); inhibit β-catenin activity.	[59,60]
TGF-β	Mainly transmits signals through Smad (e.g., Smad2/3) and non-Smad pathways. A potent pro-fibrotic factor.	Promotes fibroblast proliferation, differentiation, and excessive synthesis and deposition of ECM (especially collagen). TGF-β1/2 expression is upregulated.	Target TGF-β ligands or their receptors; inhibit Smad phosphorylation; utilize antagonism from subtypes (e.g., TGF-β3).	[120]
PI3K/Akt	Regulates cell survival, proliferation, and metabolism. Its activation is closely related to anti-apoptosis and ECM deposition.	Promotes fibroblast proliferation, migration, and inhibits apoptosis, leading to ECM accumulation.	Inhibit PI3K/Akt kinase activity; target upstream regulatory molecules (e.g., circRNA).	[125,126]
p53/p21	p53 is a main tumor suppressor gene, regulating cell cycle arrest and apoptosis; p21 is an important downstream cell cycle inhibitor of p53.	Downregulated p53 expression or functional loss leads to reduced apoptosis and uncontrolled fibroblast proliferation. High WWP1 expression further inhibits p53 activity.	Restore p53 activity or function; inhibit its negative regulators (e.g., WWP1); utilize p21-mediated cell cycle braking.	[128,129,130]
Hedgehog-GLI1	Activated during tissue repair; its abnormal activation is associated with fibrosis. Downstream transcription factor GLI1 drives fibrotic gene expression.	Pathway activity is upregulated, promoting the expression of fibrotic genes like collagen (COL1A1) and α-SMA, showing synergy with the TGF-β pathway.	Use SMO inhibitors (e.g., Vismodegib) to inhibit pathway signal transduction.	[73,131]

### 5.4. Senescent Cell Reprogramming

Given the immunoinflammatory and profibrotic microenvironment of keloids, immunotherapy is increasingly recognized as a mechanism-based adjunct to conventional scar treatments [133]. Extensive immune-fibroblast crosstalk within keloid tissue implicates cytokine-driven immune programs in sustaining KF activation and matrix overproduction [133]. Consistent with this concept, clinical reports indicate that blockade of type 2 immune signaling with dupilumab (IL-4Rα inhibition) can alleviate symptoms and stabilize lesion progression in selected keloid patients [134,135,136], supporting a pathogenic role for IL-4/IL-13-dependent pathways. Similarly, an open-label clinical trial demonstrated that the JAK inhibitor tofacitinib improved keloid severity [83], accompanied by suppression of STAT3- and Smad-associated profibrotic signaling. Moving forward, rigorous double-blind randomized controlled trials (RCTs) specifically designed for therapy-resistant keloids are urgently needed to validate these immunomodulatory interventions.

Beyond immune modulation, senescence-directed strategies provide a complementary therapeutic framework. Preclinical studies have shown that senolytic chimeric antigen receptor (CAR) T cells targeting uPAR selectively eliminate senescent cells and attenuate fibrosis [137], highlighting the feasibility of immune-mediated clearance of SASP-producing cells. Although senescence-focused gene editing approaches (e.g., targeting *p*16^INK4a^-related programs) remain preclinical in cutaneous fibrosis, they offer a conceptual basis for future precision therapies aimed at disrupting the immunosenescent niche underlying keloid persistence [137]. However, all these require large-scale controlled trials.

## 6. Future Research Direction

In January 2020, Ogawa, Rei updated and summarized the comprehensive management of keloids and proposed that pathological scars should have optimized prevention and updated algorithms based on racial circumstances [127]. Due to significant differences in skin physiology, immunology, and wound healing between animals and humans, and because keloids are unique to humans, relevant human in vitro models are needed. Lee AR et al. developed a patient-derived keloid xenograft (PDKX) model that partially recapitulates key features of human keloids, including enhanced collagen deposition and immune cell infiltration [138]. However, comprehensive comparative data validating its fidelity to human keloid biology remain limited [139]. Across keloid lesions, convergent stress inputs (including persistent inflammation, mechanical loading, and oxidative injury) are likely to stabilize the p53-p21 axis and senescence-associated transcriptional programs [20], thereby sustaining a SASP-enriched microenvironment that reinforces KF activation and immune cell recruitment. However, it is still necessary to use CRISPR to knock out SASP factors and validate the corresponding feedback loops. Within this context, redox regulation emerges as a plausible upstream control point: PRDX6, via its phospholipase A2(PLA2) activity, may amplify lipid peroxidation and oxidative stress, providing a mechanistic rationale for targeting PRDX6 to attenuate stress-induced senescence and downstream profibrotic signaling [140]. Post-transcriptional mechanisms further refine miRNA programs (e.g., miR-21 versus miR-30a-5p–BCL-2) may govern fibroblast clearance versus persistence, but require PDKX in vivo validation [141]. Together, p53-, PRDX6-, and immune-targeted interventions support rational combinations to disrupt the immunosenescent niche in keloids.

Recently, Chen-Hsiang Kuan’s team made substantial progress in skin micro-wound healing studies using wild-type C57BL/6 female mice [142], confirming that microthermal zones (MTZs) generated by fractional photothermolysis (FP) technology can significantly reduce scar formation. Collagen in the MTZs only begins to remodel 5–6 weeks after wound healing, suggesting that regulating collagen synthesis and remodeling may also exert positive effects on keloid treatment. Griffin et al. demonstrated that the ROBO2-EID1 axis attenuates fibrosis by suppressing EP300 activity [143]. Given that the embryonic origin of fibroblasts dictates their intrinsic fibrotic potential, these findings suggest that anti-scarring therapies should be tailored to specific anatomical sites [143]. miR-21 is significantly upregulated in KFs and has been shown to promote cellular survival by repressing apoptosis-related regulatory pathways [144,145], thereby contributing to KF persistence and fibrotic progression. By contrast, miR-30a-5p is downregulated in KFs and negatively modulates cellular survival by directly targeting the anti-apoptotic protein BCL2, enhancing apoptosis and suppressing proliferation [146]. Notably, trichostatin A(TSA)-mediated restoration of miR-30a-5p expression has been shown to induce apoptosis in KFs via direct BCL2 inhibition [146], thereby uncovering a microRNA-mediated regulatory axis that governs KF survival in keloids. Recent research has shown that the replicative senescence fibroblast model (FB-P30) closely resembles aged fibroblasts (FB-E) at the transcriptional level and is highly correlated with keloid-derived fibroblasts [64], indicating its utility as an in vitro model for studying the link between aging and keloid pathogenesis. As summarized in our previous work [1,3], this field remains in its early stages. Clinical translation will rely on identifying actionable senescence targets, optimizing therapeutic timing and local delivery, and validating reliable biomarkers in clinically relevant models.

## 7. Conclusions

In summary, cellular senescence provides a critical framework for elucidating keloid pathogenesis, which is driven by stress signals, redox imbalance, and immune-stromal crosstalk. This paradigm highlights the therapeutic potential of senolytics (to eliminate senescent cells) and senomorphics (to suppress their harmful secretory phenotype). However, their efficacy remains to be confirmed in RCTs.

Despite promising preclinical results, a major clinical challenge remains: delivering drugs (e.g., miRNAs, senolytics) into the dense keloid matrix. Overcoming this requires advanced local delivery systems, including exosomes, lipid nanoparticles (LNPs), and dissolvable microneedles. Notably, the clinical efficacy of local chemotherapeutics (e.g., pingyangmycin) likely derives from their ability to gradually modulate this senescent phenotype.

Although senescence-targeting therapies have shown promise in reducing fibrosis in keloid cells and animal models, their clinical application remains unclear. Given the complex nature of keloids, future research should focus on identifying key senescence pathways, enhancing treatment specificity, and evaluating efficacy in human-relevant models prior to clinical trials. To move from bench to bedside, future research must prioritize three goals. First, precisely define actionable senescence targets and reliable biomarkers using human-relevant models. Second, optimize cell-specific drug delivery and the therapeutic time window. Finally, validate safety and efficacy through rigorous clinical trials, focusing on strict endpoints like volume reduction and recurrence rates.

## Figures and Tables

**Table 1:** Molecular Markers of Cellular Senescence in Keloids.

Marker Type	Molecular Marker	Core Function	Refs.
Core Senescence Markers	SA-β-gal	Phenotypic marker; elevated activity indicates the enrichment of senescent fibroblasts, yet it exhibits weak representative characteristics in terms of inflammation, immunity and senescence	[55,92,93]
	p16^INK4a^	Cell cycle inhibitor; induces G1 arrest via CDK4/6 inhibition; core molecular marker for keloid fibroblast senescence	[55,92,93]
	p21^CIP1^	p53-regulated cell cycle inhibitor; blocks CDK2/cyclin E activity; mediates senescence-associated growth arrest in keloid fibroblasts	[55,92,93]
SASP Factors	TGF-β	Pro-fibrotic SASP component; paracrinally promotes non-senescent fibroblast proliferation and excessive ECM deposition, driving keloid expansion	[55,64,92,93]
	IL-6	Pro-inflammatory SASP factor; recruits immune cells, activates fibroblasts, and reinforces pro-fibrotic microenvironment in keloids	[34,99]
	CXCL8/IL-8	Chemotactic SASP factor; enhances fibroblast migration/proliferation and inflammatory infiltration in keloid tissues	[34,99]
	MMPs	Matrix-modifying SASP factors; mediate ECM remodeling and immune cell recruitment, amplifying keloid progression	[98]
	HMGB1	Pro-inflammatory SASP mediator; mediates paracrine senescence and immune cell recruitment to exacerbate keloid fibrosis	[96,103]
Potential Mediator	AKR1C3	Attenuates oxidative stress and enhances cell survival; hypothesized to promote senescent fibroblast persistence in keloids (needs keloid-specific validation)	[100,101,102]

## Data Availability

The original contributions presented in this study are included in the article. Further inquiries can be directed to the corresponding author.

## Abbreviations

The following abbreviations are used in this manuscript:

ECM	Extracellular matrix
SASP	Senescence-associated secretory phenotype
SRP	Senescence-resumed proliferation
QoL	Quality of life
scRNA-seq	Single-cell RNA sequencing
KFs	Keloid fibroblasts
SA-β-Gal	Senescence-associated β-galactosidase
DDR	DNA damage response
CDKs	Cyclin-dependent kinases
RB	Retinoblastoma protein
AKT/Akt	RAC-alpha serine/threonine-protein kinase
mTOR	Mechanistic target of rapamycin
NF-κB	Nuclear factor kappa-light-chain-enhancer of activated B cells
SNVs	Single nucleotide variants
cGAS-STING	Cyclic GMP-AMP synthase-stimulator of interferon genes
cGAMP	Cyclic GMP–AMP
sHLA-E	Soluble human leukocyte antigen E
YAP/TAZ	Yes-associated protein/transcriptional coactivator with PDZ-binding motif
COL1A2	Collagen type I alpha 2 chain
COL1A1	Collagen type I alpha 1 chain
HMGB1	High-mobility group box 1
AKR1C3	Aldo-keto reductase family 1 member C3
MMPs	Matrix metalloproteinases
D	Dasatinib
Q	Quercetin
siRNA	Small interfering RNA
TGF-β	Transforming growth factor-β
TGFBR2	Transforming growth factor beta receptor 2
FAK	Focal adhesion kinase
ERK	Extracellular signal-regulated kinase
SSc	Systemic sclerosis
PI3K	Phosphatidylinositol 3-kinase
circCOL5A1	Circular RNA collagen type V alpha 1 chain
IGFBP5	Insulin-like growth factor-binding protein 5
TRAF4	TNF receptor-associated factor 4
GLI1	GLI family zinc finger 1
α-SMA	Alpha-smooth muscle actin
SMO	Smoothened
IL-6	Interleukin-6
IL-8/ CXCL8	Interleukin-8
IL-4	Interleukin-4
IL-13	Interleukin-13
TNF-α	Tumor necrosis factor-alpha
VEGF	Vascular endothelial growth factor
IFN	Interferon
CDK4/6	Cyclin-dependent kinase 4/6
CDK2	Cyclin-dependent kinase 2
JAK	Janus kinase
STAT3	Signal transducer and activator of transcription 3
CAR T cells	Chimeric antigen receptor T cells
uPAR	Urokinase-type plasminogen activator receptor
RCTs	Randomized controlled trials
PDKX	Patient-derived keloid xenograft
PRDX6	Peroxiredoxin 6
PLA2	Phospholipase A2
MTZs	Microthermal zones
FP	Fractional photothermolysis
ROBO2–EID1	Roundabout guidance receptor 2–EP300 interacting inhibitor of differentiation 1
EP300	E1A binding protein p300
TSA	Trichostatin A
BCL2/Bcl-2	B-cell lymphoma 2
FB-P30	Replicative senescence fibroblast model
FB-E	Aged fibroblasts
LNPs	Lipid nanoparticles
ROS	Reactive oxygen species
